# Post-chemoradiotherapy FDG PET with qualitative interpretation criteria for outcome stratification in esophageal squamous cell carcinoma

**DOI:** 10.1371/journal.pone.0210055

**Published:** 2019-01-07

**Authors:** Yung-Cheng Huang, Shau-Hsuan Li, Hung-I Lu, Chien-Chin Hsu, Yu-Ming Wang, Wei-Che Lin, Chao-Jung Chen, Kuo-Wei Ho, Nan-Tsing Chiu

**Affiliations:** 1 Department of Nuclear Medicine, Kaohsiung Chang Gung Memorial Hospital and Chang Gung University College of Medicine, Kaohsiung, Taiwan; 2 Department of Hematology-Oncology, Kaohsiung Chang Gung Memorial Hospital and Chang Gung University College of Medicine, Kaohsiung, Taiwan; 3 Department of Thoracic and Cardiovascular Surgery, Kaohsiung Chang Gung Memorial Hospital and Chang Gung University College of Medicine, Kaohsiung, Taiwan; 4 Department of Radiation Oncology, Kaohsiung Chang Gung Memorial Hospital and Chang Gung University College of Medicine, Kaohsiung, Taiwan; 5 Department of Diagnostic Radiology, Kaohsiung Chang Gung Memorial Hospital and Chang Gung University College of Medicine, Kaohsiung, Taiwan; 6 Department of Nuclear Medicine, Yuan’s General Hospital, Kaohsiung, Taiwan; 7 Department of Health Business Administration, Meiho University, Pingtung, Taiwan; 8 Department of Nuclear Medicine, Chang Gung Memorial Hospital, Chiayi, Taiwan; 9 Department of Nuclear Medicine, National Cheng Kung University Hospital, College of Medicine, National Cheng Kung University, Tainan, Taiwan; Technische Universitat Munchen, GERMANY

## Abstract

**Objectives:**

Post-chemoradiotherapy (CRT) FDG PET is a useful prognosticator of esophageal cancer. However, debate on the diverse criteria of previous publications preclude worldwide multicenter comparisons, and even a universal practice guide. We aimed to validate a simple qualitative interpretation criterion of post-CRT FDG PET for outcome stratification and compare it with other criteria.

**Methods:**

The post-CRT FDG PET of 114 patients with esophageal squamous cell carcinoma (ESCC) were independently interpreted using a qualitative 4-point scale (Qual_4PS_) that identified focal esophageal FDG uptake greater than liver uptake as residual tumor. Cohen’s κ coefficient (κ) was used to measure interobserver agreement of Qual_4PS_. The Kaplan-Meier method and Cox proportional hazards regression analyses were used for survival analysis. Other criteria included a different qualitative approach (Qual_BK_), maximal standardized uptake values (SUV_max3.4_, SUV_max2.5_), relative change of SUV_max_ between pre- and post-CRT FDG PET (ΔSUV_max_), mean standardized uptake values (SUV_mean_), metabolic volume (MV) and total lesion glycolysis (TLG).

**Results:**

Overall interobserver agreement on the Qual_4PS_ criterion was excellent (κ: 0.95). Except the Qual_BK_, SUV_max2.5_, and TLG, all the other criteria were significant predictors for overall survival (OS). Multivariable analysis showed only Qual_4PS_ (HR: 15.41; *P* = 0.005) and AJCC stage (HR: 2.47; *P* = 0.007) were significant independent variables. The 2-year OS rates of Qual_4PS_(‒) patients undergoing CRT alone (68.4%) and patients undergoing trimodality therapy (62.5%) were not significant different, but the 2-year OS rates of Qual_4PS_(+) patients undergoing CRT alone (10.0%) were significantly lower than in patients undergoing trimodality therapy (42.1%).

**Conclusions:**

The Qual_4PS_ criterion is reproducible for assessing the response of ESCC to CRT, and valuable for predicting survival. It may add value to response-adapted treatment for ESCC patients, and help to decide whether surgery is warranted after CRT.

## Introduction

Esophageal cancer is the sixth leading cause of cancer-related mortality worldwide, and the 5-year survival rate rarely exceeds 40% [1]. Most patients with esophageal cancer have advanced disease at the initial diagnosis, and are treated with neoadjuvant chemoradiotherapy (CRT) as the standard therapy [2]. A robust stratification of patient responses to CRT based on non-invasive tools has not yet been well developed. After neoadjuvant treatment, neither clinical parameters nor endoscopic ultrasonography or CT scans can reliably predict outcome. The post-CRT FDG PET, however, has emerged as a promising predictor of long-term survival, and it can be used to tailor individualized treatment for poor responders after neoadjuvant treatment [3]. Patients whose FDG PET results were a complete response might not benefit from added resection given their excellent outcomes without resection [4]. One study [5] reported that the pooled hazard ratio (HR) for a complete metabolic response (CMR) versus no response for OS was 0.51 (95% confidence interval [CI], 0.40–0.64) and for disease-free survival was 0.47 (95% CI, 0.38–0.57), respectively. Despite its utility for predicting outcomes, the lack of uniform and reliable criteria for post-CRT FDG PET interpretation appears to be the major drawback to using the reported criteria universally. Methods to improve the predictive value of PET include a qualitative approach, e.g., comparing the tumors with healthy surrounding tissue [6]; and quantitative approaches, e.g., comparing standardized uptake values (SUVs) with reported optimum SUV cut-off values, which vary from 2.5 to 4.5 [4, 7, 8], or comparing the relative reduction in SUV between pre- and post-CRT FDG PET (ΔSUV) with reported optimum cut-off values, which vary from 35% to 70% [9–11]. Wide ranges of sensitivities and specificities have been reported. The variations appear to depend upon the different sets of criteria—which are a matter of ongoing debate—used for FDG PET interpretation. Using a qualitative interpretative criterion for response assessment of FDG PET is well established and internationally recognized as the standard of care in FDG-avid lymphoma (referred to as Deauville criteria) [12] and useful in other malignancies including head and neck cancer [13], lung cancer [14] and cervical cancer [15]. Similar harmonization guidelines for interpretive criteria are needed for esophageal cancer to compare results from different studies, to perform multicenter trials, and to assist clinical practice in different sites. We developed a simple, qualitative, interpretive criterion of FDG PET to assess esophageal cancer therapy. We validated its reader reproducibility, determined its value for predicting survival, and compared it with other visual-based and quantitative SUV-based assessment criteria.

## Materials and methods

### Inclusion and exclusion criteria

Inclusion criteria for the study were (a) histopathology-confirmed ESCC between January 2011 through December 2014, and (b) having undergone a post-therapy assessment FDG PET after the patient had completed CRT at our hospital. Exclusion criteria were (a) prior treatment for ESCC, (b) a history of other malignancies, or (c) post-therapy assessment FDG PET done more than 6 months after the patient had completed CRT. The Institutional Review Board approved this retrospective study (IRB #: 201700267B0).

### Treatment and follow-up

The CRT consisted primarily of two cycles of 5-fluorouracil/cisplatin-based chemotherapy and thoracic radiation (42–66 Gy). Trimodality therapy included a post-CRT esophagectomy, which was usually scheduled 2–4 months after the patient had completed CRT. The 7th American Joint Committee on Cancer (AJCC) staging system was used to evaluate all patients, and all were followed-up until September 2016 or until death.

### FDG PET imaging and analysis

FDG PET scans (Discovery ST PET/CT system; GE Healthcare, Waukesha, WI, USA) were principally begun one hour after the patients, who had fasted for at least 6 hours, had been injected with 370–555 MBq of FDG. Unenhanced CT scans were acquired first for attenuation correction and imaging fusion, and then PET scans (5 min/bed) from the skull to the mid-thigh were done. The PET images were reconstructed to a resolution of 5.47 × 5.47 × 3.27 mm using an ordered subsets expectation maximization algorithm. The reconstructed images were displayed in transaxial, coronal, and sagittal planes, and as a maximum intensity projection for interpretation.

For each PET dataset, the SUV_max_ was defined as the highest SUV within hypermetabolic tumor boundaries. The SUV_max_ reduction rate, i.e., the percentage reduction of the primary tumors’ SUV_max_ from pre-CRT FDG PET to post-CRT FDG PET, was calculated using the formula:

$$\Delta {\text{SUV}}_{\text{max}}\left(\%\right)=\frac{\left({\text{pre-CRT}\;\text{SUV}}_{\text{max}}-{\text{post-CRT}\;\text{SUV}}_{\text{max}}\right)}{{\text{pre-CRT}\;\text{SUV}}_{\text{max}}}\times 100$$

Qualitatively, the post-CRT FDG PET was scored using the Qual_4PS_ qualitative 4-point scale on the esophageal tumor.

- Score 1: No detectable focal uptake.
- Score 2: Focal FDG uptake greater than that in the surrounding tissue or in mediastinal blood pool, but not greater than that of the liver.
- Score 3: Diffuse FDG uptake greater than that in the mediastinal blood pool up to marginally greater than that of the liver.
- Score 4: Focal FDG uptake substantially greater than that of liver.

**Fig 1:**
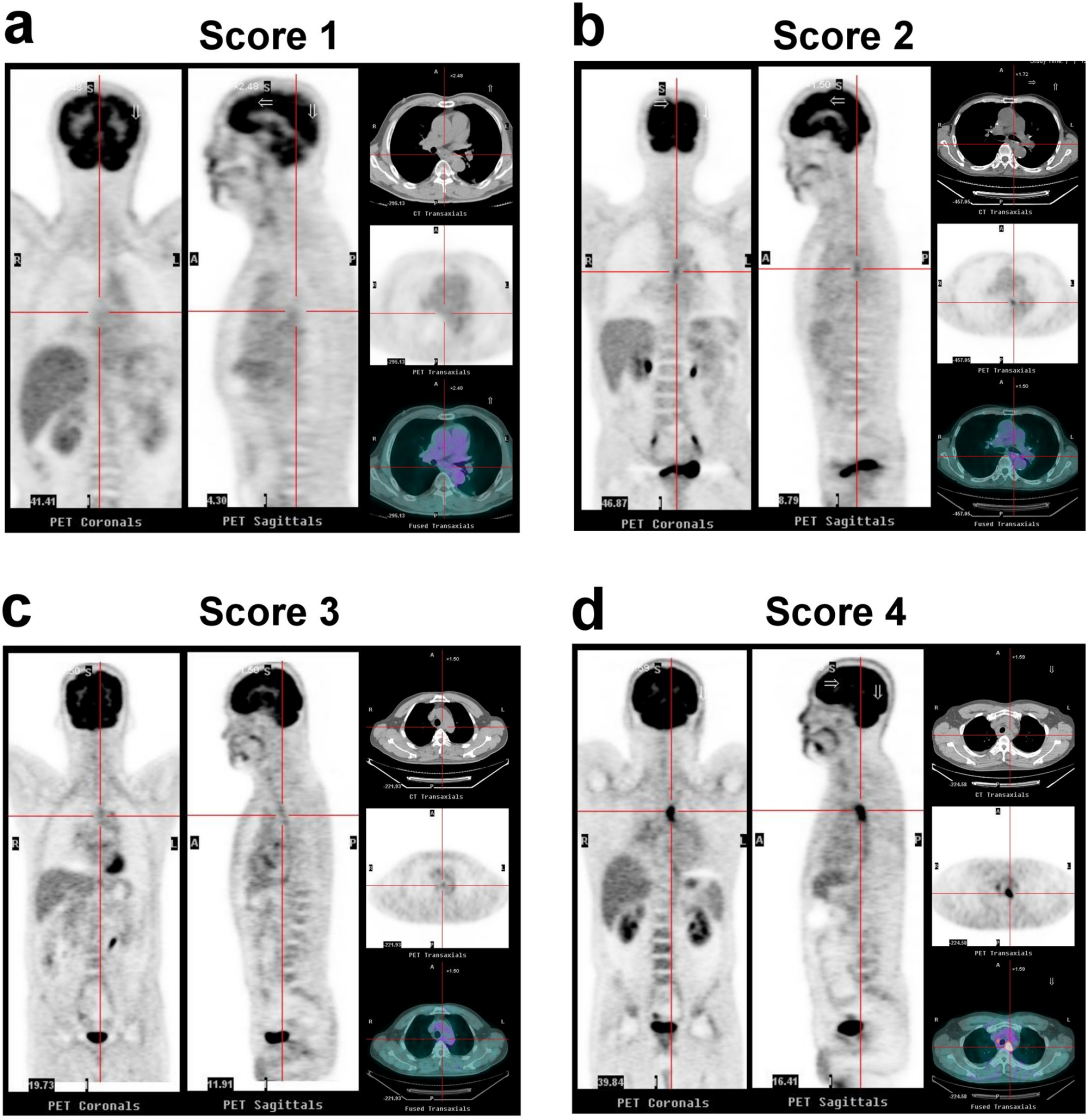
FDG PET/CT transaxial and sagittal images of four representative patients with Qual_4PS_ scores of 1–4. (a) Score 1: no detectable focal uptake; (b) Score 2: focal FDG uptake greater than that in the surrounding tissue or in the mediastinal blood pool, but not greater than that of the liver; (c) Score 3: diffuse FDG uptake greater than that in the mediastinal blood pool up to marginally greater than that of the liver, and suggestive of esophagitis; (d) Score 4: focal FDG uptake substantially greater than that of liver.

Based on the Qual_4PS_, the post-CRT FDG PET tumor assessments were grouped as positive (+) or negative (‒). Scores 1 (CMR), 2 (likely CMR), and 3 (likely post-radiation inflammation) were considered negative for a residual tumor [Qual_4PS_(‒)]. Score 4 (likely viable tumor) was considered positive for a residual tumor [Qual_4PS_(+)]. Representative examples are shown in Fig 1.

The FDG PET/CT images were retrieved from a picture archiving and communication system and were read by five experienced—25 years (NTC), 11 years (CCH), 10 years (YCH), 8 years (CJC), and 5 years (KWH)—board-certified nuclear medicine physicians at four hospitals. Blinded to patient histories and outcomes, the reviewers scored the scans independently to determine whether the reporting system would be reproducible between observers across different institutions. The final consensus result of negative or positive for a residual tumor was assigned at least 3 reviewers achieved a common agreement.

To compare the predictive value of each set of criteria, eight other criteria were also used. Qual_BK_ used the same 4-point scale, but the cut-off level was changed to surrounding background uptake [6] to make Scores 1 and 3 negative and to make Scores 2 and 4 positive. SUV_max3.4_ [16] and SUV_max2.5_ [7] were different SUV_max_ cut-offs in the primary tumor uptake on post-CRT FDG PET scans. ΔSUV_max71.6%_ and ΔSUV_max50%_ [11] used relative reduction of SUV_max_ cut-off values in the primary tumor between pre- and post-CRT FDG PET scans. The mean standardized uptake values (SUV_mean_), metabolic volume (MV), and total lesion glycolysis (TLG = SUV_mean_ × MV) were also extracted for each primary lesion. The cut-off value of ΔSUV_max_ 71.6%, SUV_mean_ 2.4, MV 2.2, and TLG 4.99 was the median data of the primary tumor in our patients. The 9 criteria stratified patients into good-responders and poor-responders.

Association between survival outcome and FDG PET/CT imaging with negative [(Qual_4PS_(‒)] or positive [(Qual_4PS_(+)] for residual primary tumor and negative (PET-CR) or positive (PET-nonCR) for malignant disease (i.e., including metastatic lesions) were analyzed to test the ability to tailor treatment for selective surgical resection. The final consensus result of Qual_4PS_(+) was documented for PET-nonCR. Unexplained FDG-avid foci in lymph nodes or distant organs were reported positive for metastases and also documented for PET-nonCR. Exceptions included mediastinal nodal tracer uptake with calcification or high attenuation (>70 household units [HU]), or characterized by symmetric low-to-intermediate intensity FDG uptake in both pulmonary hilar regions with or without extending into the subcarinal and paratracheal nodal regions. All metastatic lesions had pathologically proved or followed-up information.

### Statistical analysis

Categorical variables were expressed as frequencies (%), and continuous variables as means ± SD or medians (IQR). The levels of agreement between five reviewers were analyzed using Cohen’s κ. OS was defined as the period from the pathologically verified ESCC to the date of the last follow-up or death of the patient from any cause. The Kaplan-Meier method was used for survival analysis, and the difference between survival curves was analyzed using a log-rank test. Univariable and multivariable Cox proportional hazards regression analyses were used to identify independent predictors of OS. SPSS 17 for Windows (SPSS Inc., Chicago, IL, USA) was used for all statistical analyses. Significance was set at *P* < 0.05.

## Results

### Patient characteristics

One hundred fourteen patients (mean age: 55.2 years; range: 32–80; 3 women and 111 men) were included in the study. The median follow-up was 33.2 months for living patients (range: 20.3–69.2 months). Most patients had an Eastern Cooperative Oncology Group (ECOG) performance status score of 1 (n = 98 [86.0%]), and most were in AJCC stage III (n = 93 [81.6%]). The mean SUV_max_ of residual tumor uptake on post-CRT FDG PET was 3.8 ± 2.4. In 68 patients (60%) with available pre-CRT FDG PET scans, the mean SUV_max_ of the pre-treatment tumor was 12.8 ± 6.7, and the evaluable median ΔSUV_max_ was 71.6% (IQR: 47.9–82.7). Forty-three patients (37.7%) underwent trimodality therapy, and 71 (62.3%) underwent CRT alone (dCRT), including 10 with a salvage esophagectomy between 190 and 595 days post-CRT. The median interval between the date of the post-CRT FDG PET and the esophagectomy was 27 days (IQR: 21–37) in the trimodality group. The time between the injection of FDG and the acquisition of PET images was 58.7 ± 6.6 minutes (range: 43–80 minutes). The demographic features of the patients are summarized in Table 1.

**Table 1:** Demographic and clinical characteristics of patients.

Characteristic	
**Age, years, mean ± SD**	55.2 ± 7.9
**Male sex** ^a^	111 (97.4%)
**ECOG performance status** ^a^	
0	3 (2.6%)
1	98 (86.0%)
2	13 (11.4%)
**AJCC 7**^**th**^ **stage** ^a^	
II	6 (5.3%)
III	93 (81.6%)
IV	15 (13.2%)
**Radiation dose** ^a^	
≤ 50 Gy	42 (36.8%)
> 50 Gy	72 (63.2%)
**Post-CRT PET/CT parameter**	
Qual_4PS_ (+) ^a^	39 (34.2%)
SUV_max_ ^b^	2.8 (2.3–4.4)
ΔSUV_max_ (%) ^b^	71.6 (47.9–82.7)
SUV_mean_ ^b^	2.4 (1.9–3.0)
MV ^b^	2.2 (1.2–4.4)
TLG ^b^	4.99 (2.53–13.67)
**CRT to PET/CT interval, days** ^b^	41 (33–53)

SD: standard deviation; dCRT: treated with CRT alone; Trimodality: CRT plus a post-CRT esophagectomy; ECOG: Eastern Cooperative Oncology Group; AJCC: American Joint Committee on Cancer; Qual_4PS_: qualitative 4-point scale with liver uptake as cut-off; SUV_max_: maximal standardized uptake value; ΔSUV_max_: percentage reduction of SUV_max_ of the primary tumor from pre- to post-CRT PET/CT; SUV_mean_: mean standardized uptake value; MV: metabolic volume; TLG: total lesion glycolysis.

^a^ Data are n (%);

^b^ Data are median (IQR).

### Agreement among reviewers

**Fig 2:**
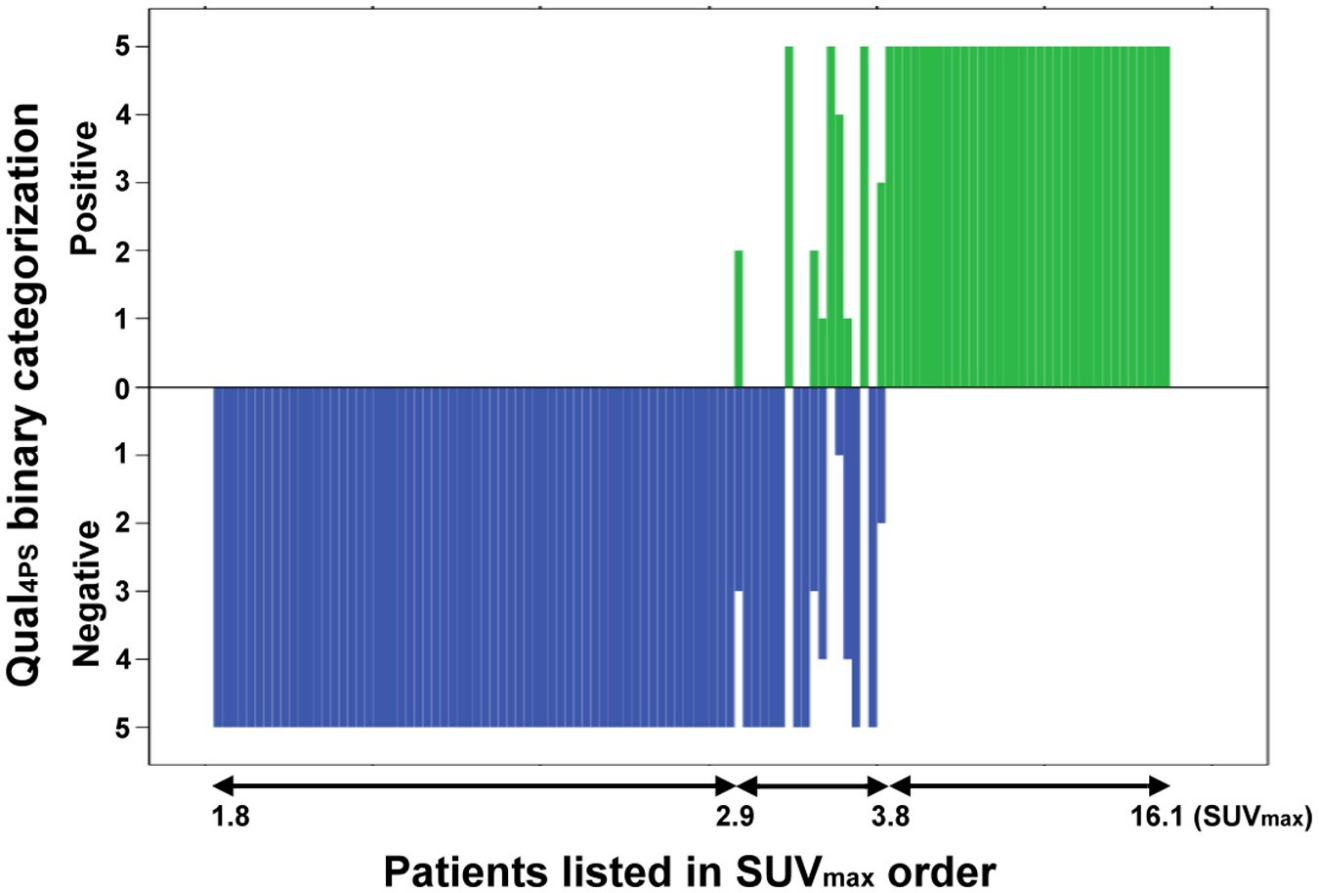
Patients listed by order of SUV_max_ plots against the number of binary categorization (positive versus negative) by five reviewers using the Qual_4PS_ criterion for post-CRT FDG PET interpretation.

In the 114 post-CRT FDG PET scans, the rates of residual tumors categorized as positive (Score 4) by the five reviewers ranged from 33.3% to 35.1%, and was 34.2% in the final consensus. The agreement of Qual_4PS_ between paired reviewers for negative versus positive results was “excellent” (Cohen’s κ: 0.923–0.961, Table 2). The overall reviewer agreement measured using Randolph’s free marginal multirater kappa was 0.95 (95%CI: 0.91–0.99). Discordant classification occurred in only 6 patients (5.3%, 3 for 3:2 and 3 for 4:1) and their tumor SUV_max_ ranged from 2.9 to 3.8. Nineteen of all study patients (16.7%) had an SUV_max_ between 2.9 and 3.8, and the discordant classification accounts for 6 of these 19 tumors (31.6%) (Fig 2).

**Table 2:** Agreement between pairs of reviewers with respect to positive versus negative PET/CT by Qual_4PS_ using Cohen’s *k*.

	**Reviewer 1**	**Reviewer 2**	**Reviewer 3**	**Reviewer 4**	**Reviewer 5**
**Reviewer 1**	1	0.942	0.923	0.943	0.961
**Reviewer 2**	0.942	1	0.942	0.923	0.941
**Reviewer 3**	0.923	0.942	1	0.943	0.961
**Reviewer 4**	0.943	0.923	0.943	1	0.942
**Reviewer 5**	0.961	0.941	0.961	0.942	1

Qual_4PS_: qualitative 4-point scale with liver uptake as cut-off

### Outcomes

**Fig 3:**
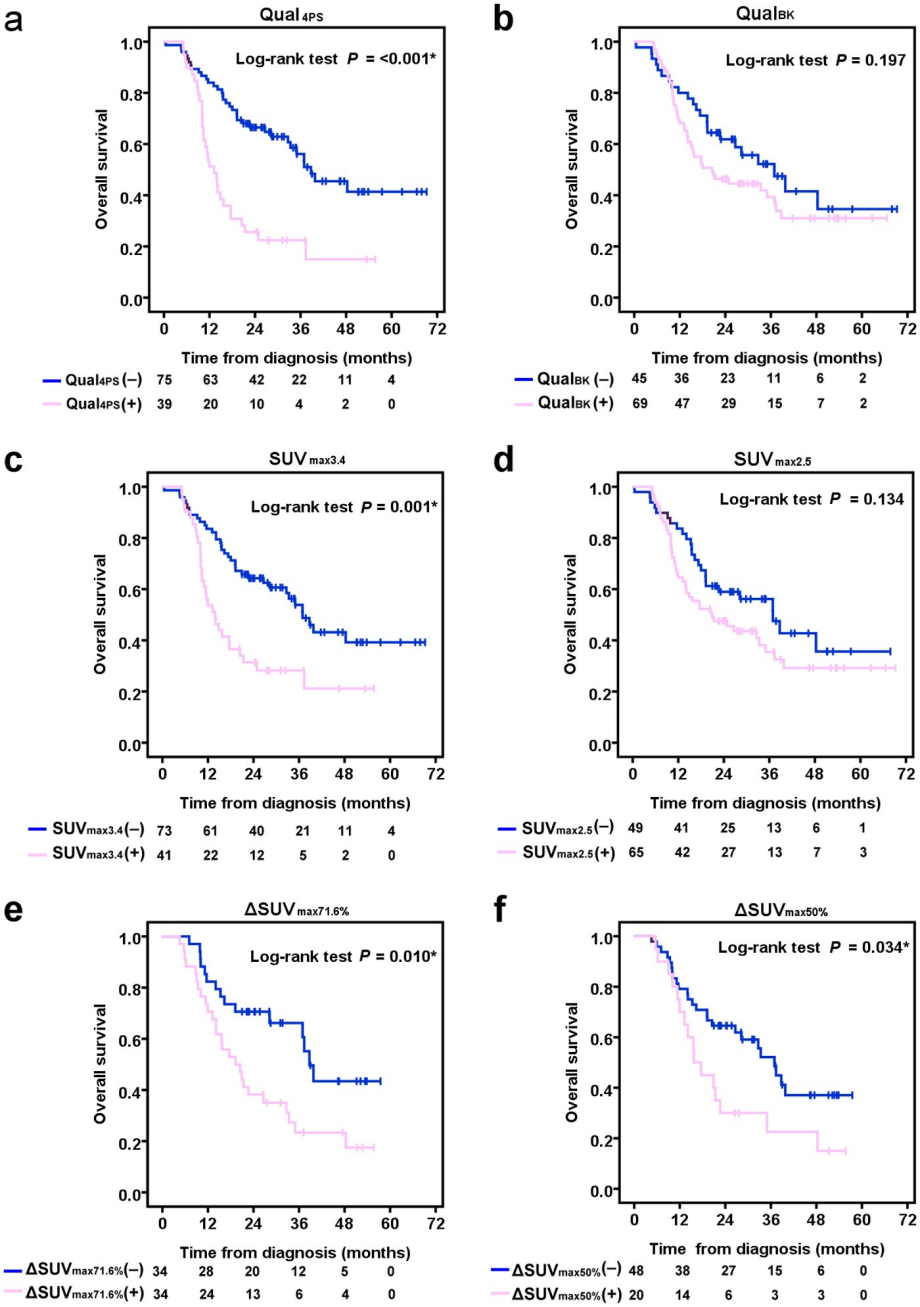
Kaplan-Meier plots of overall survival based on post-CRT FDG PET scans using (a) Qual_4PS_, (b) Qual_BK_, (c) SUV_max3.4_, (d) SUV_max2.5_, (e) ΔSUV_max71.6%_, and (f) ΔSUV_max50%_ cut-offs.

**Fig 4:**
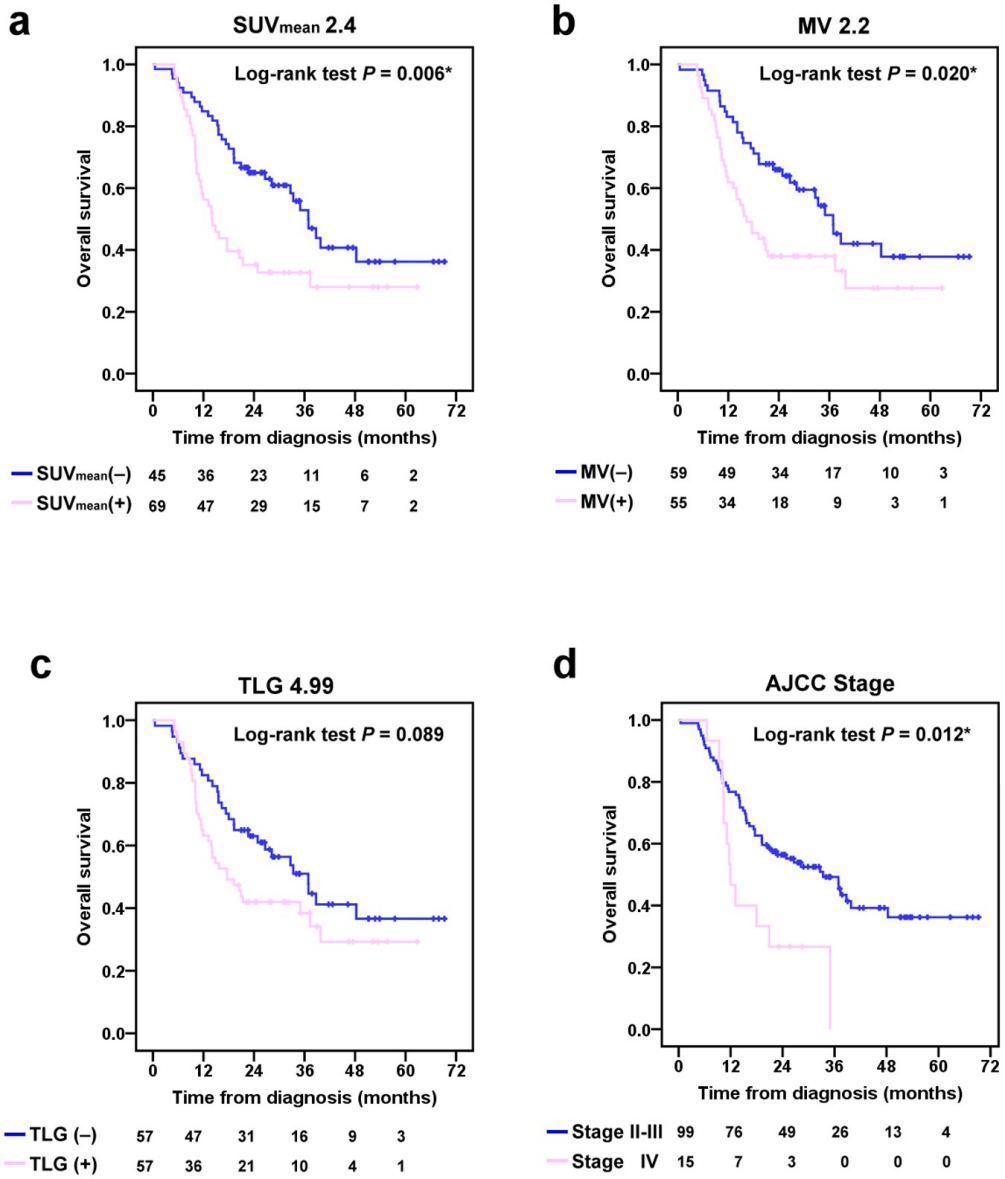
Kaplan-Meier plots of overall survival based on post-CRT FDG PET scans using (a) SUV_mean2.4_, (b) MV_2.2_, (c) TLG_4.99_ cut-offs, and (d) AJCC stage.

The median OS was 22.4 months and the 2-year OS rate was 48.1%. The 1- and 2-month postoperative mortality rates were 9.3% (4/43) and 14.0% (6/43), respectively. The Kaplan-Meier survival method and Cox proportional hazards regression analyses showed significant differences in OS between patients classified according to post-CRT FDG PET using all but the Qual_BK_, SUV_max2.5_, and TLG_4.99_ criteria (Figs 3 and 4). Univariable Cox regression analysis identified AJCC stage, and post-CRT FDG PET using the Qual_4PS_, SUV_max3.4_, ΔSUV_max71.6%_, ΔSUV_max50%_, SUV_mean2.4_, and MV_2.2_, cut-offs as significant predictors of OS (Table 3). Multivariable analysis identified only the AJCC stage (HR = 2.47; *P* = 0.007) and the post-CRT FDG PET using the Qual_4PS_ cut-off (HR = 15.41; *P* = 0.005) as significant independent variables correlated with OS.

**Table 3:** Prognostic factors for overall survival by univariable and multivariable Cox proportional hazards regression analysis.

Characteristic	Univariable					Multivariable			
						n = 68^a^		n = 114	
	1 years (%)	2 years (%)	MCLR *P*	HR (95%CI)	*P*	HR (95%CI)	*P*	HR (95%CI)	*P*
**Age**									
< 60 years (n = 82)	72.0	51.1		1					
≥ 60 years (n = 32)	75.0	56.1	0.791	1.07 (0.63–1.82)	0.791				
**Gender**									
Female (n = 3)	100	66.7		1					
Male (n = 111)	72.1	52.1	0.368	2.41 (0.33–17.36)	0.384				
**ECOG score**									
0 (n = 3)	100	66.7		1	0.817				
1 (n = 98)	71.4	51.9		1.24 (0.30–5.11)	0.765				
2 (n = 13)	76.9	53.8	0.816	0.98 (0.20–4.75)	0.982				
**BMI**									
< 23.5 (n = 77)	71.4	50.4		1					
≥ 23.5 (n = 37)	75.7	56.8	0.554	1.17 (0.69–1.98)	0.555				
**AJCC 7**^**th**^ **stage**									
II-III (n = 99)	76.8	56.4		1		1		1	
IV (n = 15)	46.7	26.7	0.012	2.23 (1.18–4.23)	0.014*	2.44 (1.03–5.73)	0.042*	2.47 (1.28–4.77)	0.007*
**Radiation dose**									
≤ 50 Gy (n = 42)	69.0	52.2		1					
> 50 Gy (n = 72)	75.0	52.8	0.657	0.98 (0.58–1.67)	0.951				
**Post-CRT PET**									
• Qual_4PS_(-), Score 1,2,3 (n = 75)	84.0	66.5		1		1		1	
Qual_4PS_(+), Score 4 (n = 39)	51.3	25.6	<0.001	2.89 (1.76–4.73)	<0.001*	6.55 (0.55–77.46)	0.136	15.41 (2.07–114.99)	0.005*
• Qual_BK_(-), Score 1 & 3 (n = 45)	80.0	61.9		1					
Qual_BK_(+), Score 2 & 4 (n = 69)	68.1	46.3	0.197	1.39 (0.84–2.32)	0.199				
• SUV_max_ ≤ 3.4 (n = 73)	83.6	64.3		1		1		1	
SUV_max_ > 3.4 (n = 41)	53.7	31.4	0.001	2.30 (1.40–3.75)	0.001*	0.54 (0.09–3.29)	0.506	0.39 (0.10–1.57)	0.186
• SUV_max_ ≤ 2.5 (n = 49)	83.7	59.0		1					
SUV_max_ > 2.5 (n = 65)	64.6	47.6	0.134	1.46 (0.89–2.40)	0.136				
• ΔSUV_max_ >71.6% (n = 34)	82.4	70.6		1		1			
ΔSUV_max_ ≤ 71.6% (n = 34)	70.6	38.2	0.010	2.27 (1.20–4.30)	0.012*	1.73 (0.88–3.41)	0.111		
• ΔSUV_max_ > 50% (n = 48)	79.2	64.6		1					
ΔSUV_max_ ≤ 50% (n = 20)	70.0	30.0	0.034	1.96 (1.04–3.68)	0.037*				
• SUV_mean_ ≤ 2.4 (n = 66)	84.8	65.0		1		1		1	
SUV_mean_ > 2.4 (n = 48)	56.2	35.2	0.006	1.96 (1.21–3.19)	0.007*	0.65 (0.07–5.64)	0.693	0.42 (0.09–1.92)	0.264
• MV ≤ 2.2 (n = 59)	83.1	66.0		1		1		1	
MV > 2.2 (n = 55)	61.8	37.9	0.020	1.77 (1.09–2.88)	0.021*	0.78 (0.34–1.80)	0.565	1.50 (0.81–2.81)	0.200
• TLG ≤ 4.99 (n = 57)	82.5	63.0		1					
TLG > 4.99 (n = 57)	63.2	41.9	0.089	1.52 (0.93–2.47)	0.091				
**Treatment protocol**									
dCRT (n = 71)	73.2	52.0		1					
Trimodality (n = 43)	72.1	53.4	0.946	0.98 (0.60–1.62)	0.946				

MCLR: Mantel-Cox log-rank; HR: hazard ratio; CI: confidence interval; ECOG: Eastern Coope0.946rative Oncology Group; BMI: body mass index; AJCC: American Joint Committee on Cancer; Qual_4PS_: qualitative 4-point scale with liver uptake as cut-off; Qual_BK_: qualitative 4-point scale with surrounding background uptake as cut-off; SUV_max_: maximal standardized uptake value; ΔSUV_max_: percentage reduction of SUV_max_ of the primary tumor from pre- to post-CRT PET; SUV_mean_: mean standardized uptake value; MV: metabolic volume; TLG: total lesion glycolysis; dCRT: treated with CRT alone; Trimodality: CRT plus a post-CRT esophagectomy.

^a^ Sixty-eight of total 114 patients had pre-CRT FDG PET scans and evaluable ΔSUV_max._

**Fig 5:**
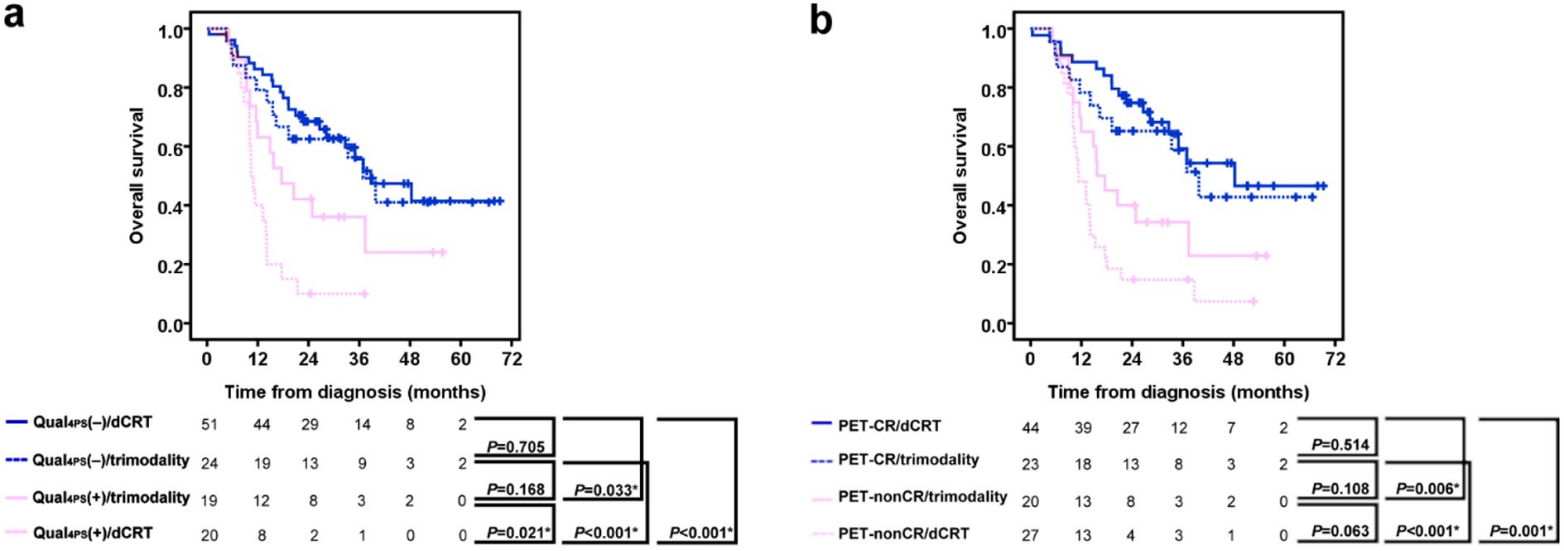
Kaplan-Meier plots of overall survival for subgrouping patients according to therapeutic management: dCRT versus trimodality and post-CRT FDG PET results based on (a) Qual_4PS_ and (b) PET-CR.

Based on FDG PET classification according to the Qual_4PS_ interpretation criteria [Qual_4PS_(‒) or Qual_4PS_(+)] and therapeutic management (dCRT or trimodality), our patients could be divided into four distinct subgroups with different OS rate. The 2-year OS rates were 68.4% for the Qual_4PS_(‒)/dCRT group, 62.5% for the Qual_4PS_(‒)/trimodality group, 42.1% for the Qual_4PS_(+)/trimodality group, and 10.0% for the Qual_4PS_(+)/dCRT group. The Qual_4PS_(‒)/dCRT and Qual_4PS_(‒)/trimodality had equivalent OS rates. The Qual_4PS_(‒)/trimodality group had a nonsignificantly higher survival rate than did the Qual_4PS_(+)/trimodality group. The Qual_4PS_(+)/dCRT group had a significantly lower survival rate than did the other three groups (Fig 5a). These data indicated that dCRT or trimodality resulted in no significant difference of 2-year OS for patients with Qual_4PS_(‒) while trimodality might be a better choice for patients with Qual_4PS_(+). Using PET-CR based on Qual_4PS_ for FDG PET scan to subgrouping patients, the 2-year OS rates were 74.8% for the PET-CR/dCRT group, 65.2% for the PET-CR/trimodality group, 40.0% for the PET-nonCR/trimodality group, and 14.8% for the PET-nonCR/dCRT group (Fig 5b). After excluding patients with stage IV, similar differences in survival between subgroups were also obtained (S1 Fig).

## Discussion

We found that using the proposed Qual_4PS_ qualitative interpretation criterion for post-CRT FDG PET to assess treatment response of ESCC provided good predictive value for survival outcome and yielded excellent interobserver agreement between reviewers from different hospitals. Additionally, it might offer a guide for deciding on post-CRT surgery. FDG PET has been evaluated to optimize monitoring therapeutic response of ESCC and other malignancies. Widely available and easy-to-use SUV is the method of choice in most studies. However, the reliability of SUV measurement is affected by many factors, such as inter-scanner variability, calibration errors, image acquisition and reconstruction parameters, attenuation correction, scatter correction, respiratory motion, and the partial volume effect, all of which make proper comparisons between different cohorts problematic [17]. One aim of this study was to determine whether a qualitative scoring system is practical and sufficiently robust to enable standardization of reporting across different hospitals. The major problems of a qualitative visual interpretation are the necessity of suitable criteria for interpretation and of reproducibility between different observers. Using the liver cut-off as a reference target seemed appropriate for post-therapy FDG PET interpretation. After they had studied a serial example of the scoring system (Fig 1), five reviewers from four different hospitals showed excellent agreement (Randolph’s kappa: 0.95).

Various analytic models have been published for predicting outcome and optimal discrimination between responders and nonresponders by defining (a) a cut-off level for the residual tumor FDG uptake on the post-therapy scan, or (b) a percentage decrease of SUV level between pre- and post-therapy scans. There is, however, no consensus about which post-therapy FDG PET cut-off criteria are the best predictors of the outcomes of ESCC patients or which most accurately identify patients who benefitted from surgery. Jeong et al. [6] qualitatively defined PET-CR as a decreased FDG uptake to a level indistinguishable from that of the surrounding normal tissue. Moreover, SUV_max_ levels ≤ 3.0 [4] and < 2.5 [7] have been used quantitatively for PET-CR. All of them verified that post-therapy PET-CR was a significant independent predictor of improved outcomes for CRT. There has been growing interest in using ΔSUV as a robust method of measuring metabolic response for predicting good and poor outcomes [18]. A large part of these studies chose the median value of their cohort data for the cut-off. Thus, a wide range of SUV reduction cut-offs, from 35% to 70%, have been reported [9–11]. The variance suggests a lack of standardization and might be explained by factors such as the spectrum of disease severity, and differences in the clinical features and therapy of each selected patient group. In this study, we also tested (a) the adjusted cut-off of SUV_max3.4_, because of its optimal ability to detect post-CRT viable residual tumors in our institution [16], and (b) the adjusted cut-off of ΔSUV_max_ 71.6%, which was the median reduction value in this cohort. As expected, the adjusted cut-off of SUV_max3.4_ was better than the reported SUV_max2.5_, and the adjusted cut-off of ΔSUV_max71.6%_ was better than the reported ΔSUV_max50%_ for predicting good and poor outcomes.

An optimal treatment strategy should balance improved survival with minimized therapy-related morbidity, mortality, and quality-of-life deterioration. The necessity of surgical resection after CRT remains controversial. We found equivalent survival for patients in the CRT-alone and trimodality groups, which was consistent with the results of other randomized trials in which most patients had ESCC [19, 20]. Post-CRT esophagectomies are associated with an approximately 50% postoperative morbidity rate and a 10% postoperative mortality rate [21–23]. As in this cohort, the 1- and 2-month postoperative mortality rates were 9.3% and 14.0%, respectively. A non-invasive surrogate marker after CRT is needed to indicate that additional surgery can be delayed, or even omitted, or that is can be requested. An endoscopic biopsy for pathologic responses might not be the best predictor of outcomes after CRT in esophageal cancer; the association of PET-CR with outcomes is believed to be more clinically relevant [24, 25]. Retrospective studies which evaluated the potential of PET response-adapted strategy to identify patients for whom surgery might be avoided reported that the OS of PET-CR patients treated with CRT alone were equivalent to those treated with trimodality therapy [4, 26]. In a prospective multicenter study of 43 patients, tailoring treatment based on post-CRT FDG PET scans for selective surgical resection showed promising efficacy [27]. In our study, the additional post-CRT esophagectomy significantly improved the OS of post-CRT Qual_4PS_(+) patients, but it did not significantly improve the OS of post-CRT Qual_4PS_(‒) patients. After CRT, using FDG PET with the Qual_4PS_ interpretation criterion might be useful for determining the need for additional surgery. Large randomized multicenter studies to further evaluate this organ-preserving approach of individualized therapy are still required, however. We believe that the harmonious interpretation criterion “Qual_4PS_” presented here is suitable for post-CRT FDG PET scans of future multicenter trials to identify patients who benefit from CRT and, therefore, have a favorable outcome.

The study was retrospective and thus prone to a selection bias. Our results are not sufficient enough to change routine clinical practice for all esophageal cancer and should be interpreted cautiously. Six of the 19 patients (31.6%) with an SUV_max_ between 2.9 and 3.8 were given discordant classifications by the reviewers. For tumor uptake within the relatively challenging SUV_max_ range, we recommend that multiple reviewers be required for reaching a comprehensive consensus. It is necessary to include prospective trials that evaluate FDG PET response based on the Qual_4PS_ criterion to predict outcomes of esophageal cancer and that is embedded in a randomized treatment algorithm.

## Conclusions

The proposed Qual_4PS_ interpretation criterion of FDG PET as therapy assessment for ESCC has excellent interobserver agreement and provides good predictive value for survival outcome. It is comparable to, and even better than quantitative criteria with different cut-offs. It can provide important information about which patients will benefit from an esophagectomy after CRT.

## Supporting information

S1 Fig The Kaplan-Meier plots of overall survival for subgrouping patients without stage IV disease according to therapeutic management: dCRT versus trimodality and post-CRT FDG PET results based on (a) Qual_4PS_ and (b) PET-CR. (TIF)
