# Supplementary figures and images for: Post-chemoradiotherapy FDG PET with qualitative interpretation criteria for outcome stratification in esophageal squamous cell carcinoma
Source: PLoS One. 2019 Jan 7;14(1):e0210055. doi: 10.1371/journal.pone.0210055 (PMC6322736; doi:10.1371/journal.pone.0210055)

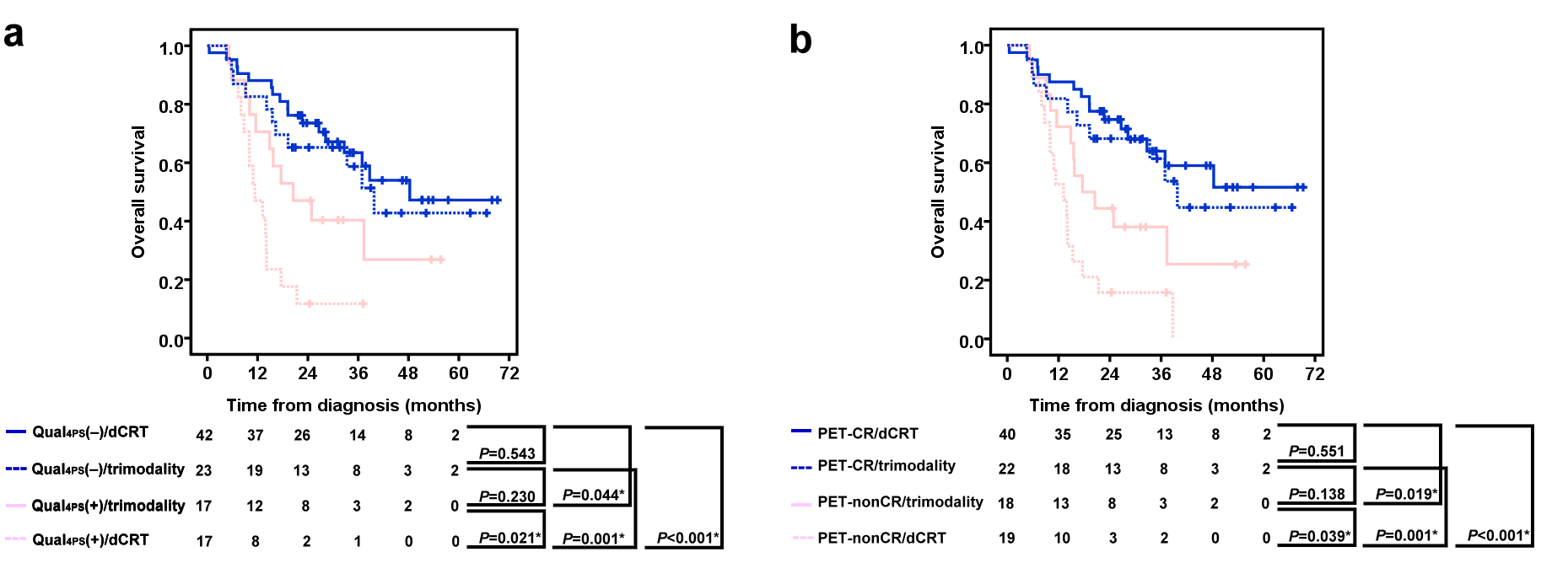

Supplement: S1 Fig — (TIF) [file pone.0210055.s001.tif]
